# Solution structure of human MBD1 CXXC1

**DOI:** 10.1007/s10858-015-9986-8

**Published:** 2015-09-09

**Authors:** Ross Thomson, Brian O. Smith

**Affiliations:** Institute of Molecular Cell and Systems Biology, College of Medical, Veterinary and Life Sciences, Joseph Black Building, University of Glasgow, Glasgow, G12 8QQ UK

## Biological content

Methylation of a CpG dinucleotide at cytosine C5 (mCpG) is a major modification in vertebrate genomes associated with epigenetic gene silencing. CpG methylation recruits proteins which specifically recognise this motif and these methylated DNA binding proteins then recruit enzymes which chemically and physically alter chromatin, inducing transcriptional repression. Although most CpG motifs are methylated, short (500–2000 bp) CG-rich regions, known as CpG islands (CGIs), found within ~60 % of human gene promoters remain non-methylated (Bird 2002). How these CGIs contribute to epigentic regulation is an area of continuing research.

To investigate the possibility that proteins are targeted to these CGIs, Voo et al. (2000) used a ligand screen to identify proteins that bind non-methylated CpGs. They identified a protein, CFP1 (formerly CGBP) that contains a cysteine rich zinc finger–CXXC (ZF-CXXC) domain. Since this discovery, several more CXXC containing proteins have been identified as reviewed recently by Long et al. (2013). CXXC domains can be differentiated into three groups, type 1 which bind CpG and contain the KFGG motif, type 2 which do not bind CpG and lack the KFGG motif, and type 3 which lack the extended loop of types 1 and 2 but do bind to CpG (Fig. 3a). The conserved CXXCXXCX_4/5_CXXCXXC and CXXRC motifs that provide the amino acid residues that coordinate the two zinc ions means that the main domain architecture, despite sequence variation between the three types, is similar in all CXXC domains.

CXXC domains are found in various chromatin binding proteins: CFP1 is a member of the SETDB1 complex responsible for H3K4 methylation; MLL protein is also a H3K4 methyltrasferase; KDM2A and KDM2B are H3K36 demethylase enzymes. DNMT1 and MBD1 are unique amongst CXXC domain containing proteins in that they can also bind hemi-methylated and methylated CpG respectively as well as non-methylated CpG. As well as the methylated DNA binding domain, MBD1 also contains a transcriptional repressor domain (TRD) and depending on splice variant, 2 or 3 CXXC domains. The TRD is capable of transcriptional repression independently of the MBD domain, and MBD1 induces transcriptional repression by recruiting the histone H3-K9 methyl transferase enzyme SETDB1. After DNA replication, this histone methylation is heritably maintained due to MBD1 remaining at the replication fork through its interaction with chromatin assembly factor 1. Once replication has occurred, DNMT1 activity fully methylates hemimethylated CpG motifs allowing MBD1 to rebind to the methyl-CpG (Sarraf and Stancheva 2004).

There are several published structures of type 1 (Allen et al. 2006; Cierpicki et al. 2010; Song et al. 2011; Xu et al. 2011) and type 3 (Xu et al. 2012) (unpublished data, PDB depositions 4BBQ, 4O64) CXXC domains with and without DNA bound that have revealed how the CXXC domain interacts with CpG DNA. The CXXC domains bind perpendicular to the DNA helical axis, with their DNA binding loops packed into the major groove. The type 1 DNA binding motif consists of three residues, in the case of hMLL, ^1185^KKQ^1187^, which hydrogen bond to the CpG sequence. The main chain carbonyls of K1185 and K1186 form hydrogen bonds to the N4 amines of the cytosine bases while the side chain amides of K1186 and Q1187 form hydrogen bonds to the guanines (Cierpicki et al. 2010). The first DNA binding residue is not strongly conserved amongst the type 1 CXXC domains, unlike the second and third which are either KQ or, in the case of CFP1 and MBD1 CXXC3, RQ. The type 3 domain DNA binding interface is similar and consists of XHQ but CXXC1 lacks these positively charged residues and CXXC2 only has one. The CXXC domains are unable to bind to methyl-CpG due to a steric clash between the methyl group and the protein backbone that prevents hydrogen bonding to the DNA bases.

Although MBD1 can contain up to 3 CXXC domains, only the third, type 1, CXXC domain is able to bind a CpG motif. MBD1 CXXC1 and 2 are type 2 domains and although similar to the type 1 in that they have an extended loop between the sixth and seventh cysteine they lack the conserved KFGG sequence and DNA binding motif. In this study we used NMR to determine the solution structure of the type 2 MBD1 CXXC1 domain in order to compare it to the published type 1 CXXC structures. Using backbone dynamics we also investigated whether the lack of the KFGG motif in MBD1 CXXC1 affects the rigidity of the structure.

## Methods and results

### Expression and purification of hMBD1 CXXC1

MBD1 CXXC1 domain, residues 167–222, was amplified from the EST clone, accession number CF552871 (Geneservice Ltd ID 30529682) using the PCR primers: hMBD1 CXXC1 forward 5′ GGGATCCGAGCAGAGAATGTTTAAG 3′; hMBD1 CXXC1 reverse 5′ CTCGAGTCAGCTCCTTTCCACAATC 3′. The plasmid pGEX-6p1 (GE healthcare) containing the CXXC domain from hMBD1 was over expressed in *E. coli* Tuner™ DE3 cells (Novagen) as an N-terminal glutathione-S transferase (GST) fusion. Protein expression was induced (OD_600_ 0.6–0.8) with 0.3 mM IPTG at 13 °C overnight. Cells were harvested and resuspended in phosphate buffered saline solution (PBS) pH 7.3 (10 ml per 1 l of culture). Bacteria were disrupted using BugBuster™ (Novagen) and DNA digested with Benzonase^**®**^ nuclease (Novagen). The protein was purified by affinity chromatography using glutathione Sepharose 4 Fast Flow resin and the tag removed on-column by PreScission Protease. To remove EDTA from the PreScission protease it was first dialysed against 50 mM Tris–HCl (pH 8.0), 150 mM NaCl, 1 mM DTT using a Thermo Scientific Slide-A-Lyzer Dialysis Cassette, 7000 molecular weight cut off (MWCO). Protein samples were buffer exchanged into 5 mM deuterated imidazole, 250 mM NaCl, 1 mM deuterated DTT and concentrated to a final volume of 570 µl using a Vivaspin 20 5000 MWCO centrifugal concentrator (Sartorius Stedim). For isotopically labelled protein, the bacteria were cultured using M9 minimal containing 1 g/l ^15^N-ammonium chloride.

### NMR spectroscopy and NMR Resonance assignment

NMR data were recorded on Bruker AVANCE 600 MHz and 800 MHz spectrometers equipped with 5 mm TCI cryoprobes using 1 mM protein samples. 2D ^1^H TOCSY, 2D ^1^H NOESY and 2D ^1^H COSY spectra were recorded on unlabelled protein in samples containing 5 % D_2_O and 100 % D_2_O. 2D ^15^N HSQC, 3D ^15^N HSQC-TOCSY, 3D ^15^N HSQC-NOESY, HNHA and HNHB were recorded using ^15^N labelled protein. All NOESY experiments used for structure calculation were recorded with a mixing time of 100 ms and at a temperature of 293 K. Data was processed with AZARA and analyzed with CCPN analysis software (Vranken et al. 2005). Maximum entropy reconstruction (Barna et al. 1987) was used to enhance the resolution of indirect dimensions in the 3D NOESY experiments.

Using the data obtained, 94.5 % of the backbone and 86.5 % of the side chain resonances where assigned for residues 167–222. The aromatic side chain protons of the two phenylalanine residues (171, 207) were assigned by using the 2D ^1^H NOESY experiments recorded in D_2_O and in H_2_O. The chemical shift assignments have been deposited with the BMRB under accession 25312.

^15^N *T*_*1*_, *T*_*2*_ and heteronuclear-NOE relaxation data were recorded at 600.13 MHz (^1^H) and 293 K and the data were analyzed according to the Lipari–Szabo model-free formalism using the program FAST-model free (Cole and Loria 2003). For *T*_*1*_ measurements, data was recorded with relaxation delays of 0.1, 1, 2, 4 s and for *T*_*2*_ measurements, data was recoded with relaxation delays of 17, 34, 68, 102, 136, 237 ms. The recycle delay for both *T*_*1*_ and *T*_*2*_ experiments was 2.2 s. Heteronuclear NOE values were determined from a pair of spectra recorded with and without proton saturation. The inter-scan relaxation delay for the heteronuclear NOE experiments was 6 s incorporating 3 s of proton saturation for the proton saturation experiment. The rotational correlation time (τ_c_) for each residue was estimated using the *r2r1_tm* script (A. G. Palmer III, www.palmer.hs.columbia.edu/software.html), which calculates τ_c_ from the ratio of R2/R1. Residues with NOE <0.6 or with an R2/R1 ratio more than one standard deviation from the mean were omitted. The molecular rotational time (τ_m_) of 5.67 ± 0.141 ns for the entire molecule was then estimated by taking the mean τ_c_ of the remaining residues.

### Structure calculation

**Fig. 1:**
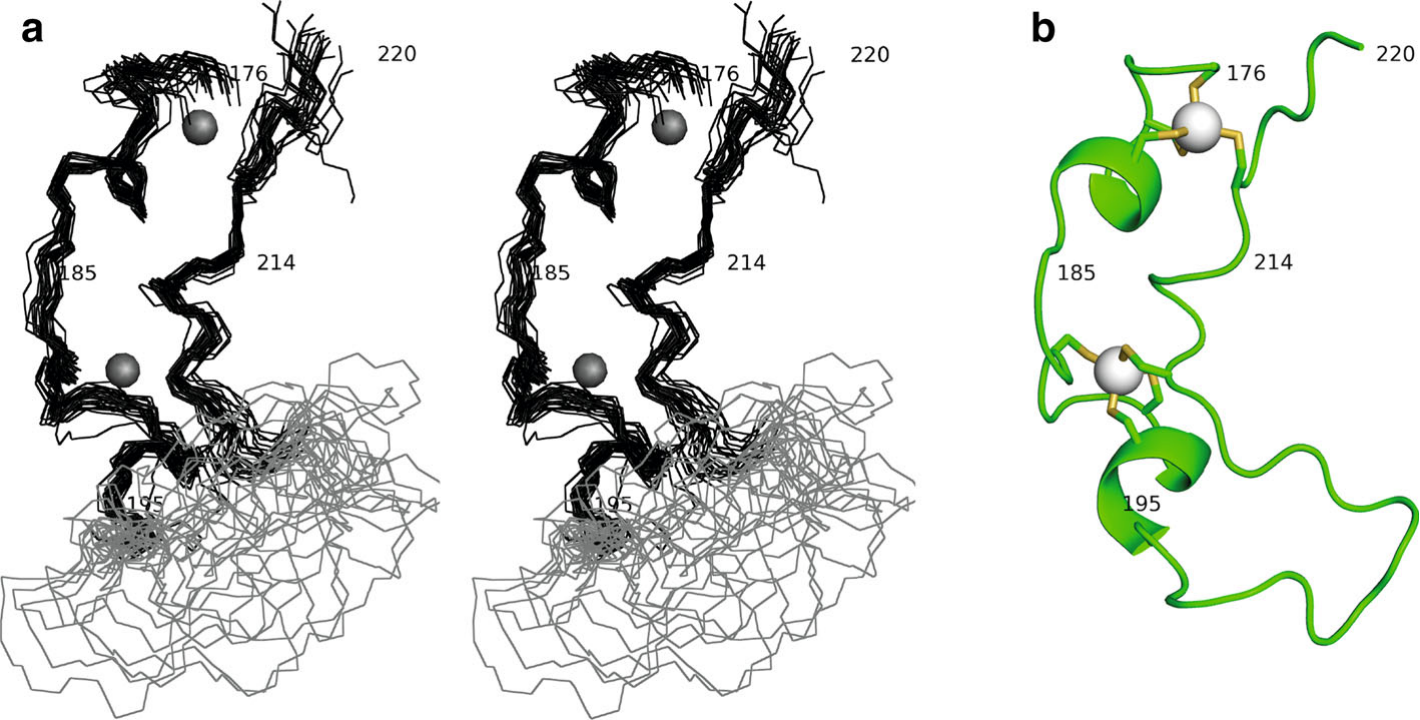
The structure of hMBD1 CXXC1. **a** A stereo representation of the ensemble of MBD1 CXXC1 (residues 176–220) structures determined by NMR spectroscopy. The backbone trace is shown and selected residues labelled with the poorly defined loop (residues 196–206) shown in grey. Zinc ions are shown as grey 1 Å spheres. **b** Cartoon view of the solution structure of the representative structure from the ensemble with zinc atoms shown as *grey spheres*, each bonded to their coordinating cluster of cysteine residues

Structure calculations were carried out using ARIA 2.3 (Ambiguous Restraints for Iterative Assignment) (Rieping et al. 2007) over 8 iterations. 24 structures were calculated in iterations 1–5, 50 in iteration 6, 100 in iteration 7 and 200 in iteration 8. 25 of the final 200 structures (Fig. 1a) were refined in a shell of water molecules with a full molecular dynamics force field incorporating electrostatics (Linge et al. 2003). Spin diffusion correction was included from iteration 3 using a distance cut off of 6 Å. In order to allow the use of spin diffusion correction during structure calculation, ARIA was used to derive distance restraints from the 3D ^15^N NOESY-HSQC and 2D ^1^H NOESY spectra. In addition to the 1327 NOE distance restraints, 38 ^3^J_HN-Hα_ (extracted from a 3D HNHA spectrum), 17 ^3^J_HN-Hβ_ restraints (derived from a 3D HNHB spectrum) and zinc co-ordination restraints for each of the cysteines (Cys 176, 179, 182 and 215 for zinc 1; Cys 188, 191, 194 and 210 for zinc 2) were also included and are summarised in Table 1. The assignment of the cysteine residues to the two zinc clusters was determined by initially calculating structures without zinc atoms or coordination restraints. In these calculations, the cysteine residues consistently clustered as indicated above. The zinc coordination restraints defined the bond lengths between the sulphur atoms of the cysteines and the zinc ion as well as the bond angles. The ensemble RMSD to the unbiased mean coordinates [determined using uwmn (Leo Caves, unpublished); uwmn implements an average distance matrix method to determine the unbiased mean structure which is then back-projected into cartesian space, and from which the ensemble RMSDs are calculated following superposition of the individual structures] for residues 175–196 and 208–217 of the water refined structures are 1.040 Å (all heavy atoms) and 0.751 Å (backbone heavy atoms) while PROCHECK-NMR (Laskowski et al. 1996) was used for validation. Greater than 90 % of the residues fall within the most favorable and additionally allowed regions. However, the contribution of unfavorable φ and ψ angles in the unstructured N- (163–174) and C- (218–222) termini as well as the poorly defined loop region (197–207) results in higher than usual number of residues in the disallowed region. If the unstructured regions are removed from the Ramachandran analysis, the percentage of residues in the disallowed regions is reduced and the proportion of residues falling within the most favorable and additionally allowed regions increases to over 97 %. The NMR restraints and coordinates of the best 20 structures were deposited in the Protein Data Bank under accession code 4D4W.

**Table 1:** Structural statistics for the 20 lowest energy structures of hMBD1 CXXC1

*NMR Dist NMR distance restraints*	
Total NOE	1327
Ambiguous	593
Intra-residue	204
Inter-residue	
Sequential (|i − j| = 1)	156
Short range (1 < |i − j| ≤ 4)	118
Long range (|i − j| ≥ 5)	115
Unambiguous	734
Intra-residue	334
Inter-residue	
Sequential (|i − j| = 1)	211
Short range (1 < |i − j| ≤ 4)	102
Long range (|i − j| ≥ 5)	87
Zinc co-ordination restraints	
S–Zn bond length	2.3 Å
Cα–Cβ–S bond angle	114.3558°
Cβ–S–Zn bond angle	109.5000°
Hβ–Cβ–S bond angle	107.9185°
S–Zn–S bond angle	109.5000°
*Structural statistics (20 structures)*	
Constraint violations per structure	
>0.5 Å (±SD)	0.10 (±0.06)
>0.3 Å (±SD)	1.35(±0.16)
> 0.1 Å (±SD)	19.05 (±0.81)
R.M.S. deviations from the ideal geometry	
Bond length (Å)	0.0038 (±0.000023)
Bond Angle (°)	0.600 (±0.05)
Improper angles (°)	1.476 (±0.04)
Dihedrals (°)	42.17 (±0.137)

Ramachandran plot analysis	% of residues^a^
Residues in most favoured regions	61.3 % (77.7 %)
Residues in additional allowed regions	32.3 % (21.0 %)
Residues in generously allowed regions	3.3 % (0.7 %)
Residues in disallowed regions	3.1 % (0.7 %)

^a^Residues from well-defined regions of the structure, 175–196 and 208–216

## Discussion and conclusions

### Overview of the solution structure of hMBD1 CXXC1

The solution structure of MBD1 CXXC1 (residues 167–222) adopts a crescent shape incorporating two zinc ions (Fig. 1b). Four cysteines provide the ligands for the coordination of each zinc ion, three from each CXXCXXC motif. The main chain loops back 180° after the second CXXCXXC motif providing the two further cysteines that complete the zinc coordination sites. The overall structure of the domain is governed by the presence of the two zinc clusters. Mutations of one of the zinc coordinating cysteines will destabilise the structure of the MLL CXXC domain (Allen et al. 2006. Cierpicki et al. 2010), MBD1 CXXC3 (Clouaire et al. 2010) and CGBP (Lee et al. 2001) suggesting the CXXCXXC motifs are absolutely required for maintaining the structure of the domain. The zinc clusters have similar structures to each other with a backbone RMSD of 0.86 Å over the CXXCXXC sequence [calculated using Superpose (Maiti et al. 2004)] and form a small helical turn between the first and second cysteines followed by a single turn of alpha helix, that includes residues 180A–183Q (cluster 1) or (192S–196L) cluster 2. Residues 197–207 lack long range NOEs and thus form a flexible, poorly defined loop that is markedly different to the type 1 CXXC domain DNA binding loop region, while residues 167–174 and 218–222 form flexible N- and C-termini respectively.

### ^15^N backbone dynamics

**Fig. 2:**
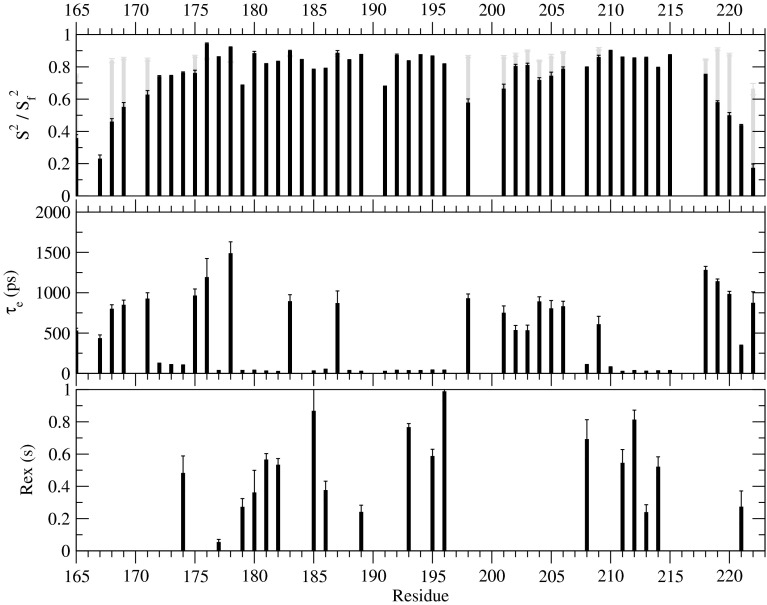
Backbone dynamics parameters of hMBD1 CXXC1. Extended Lipari–Szabo model free parameters derived from ^15^N relaxation parameters are presented for each residue where a motional model gave an acceptable fit. The overall correlation time was estimated as 5.6 ns

Fast-model free was able to assign internal dynamic models to 47 out of 56 residues (Fig. 2), 1 represented by model 1 (S^2^), 8 by model 2 (S^2^, τ_e_), 18 by model 4 (S^2^, τ_e_, R_ex_) and 20 by model 5 (S^2^, τ_e_, S_f_^2^). Five of the eight residues were not fitted to any model (190A, 197Q, 207F, 216L, 217R), while two were not assessed because their amides could not be assigned (170M, 200H) and one is a proline residue (199P).

The results show the two termini and loop region are dynamic with low S^2^ values and internal correlation times on the pico- to nano-second time scale. The loop region is further characterised by requiring the S_2_^f^ function to fit the internal motion to a model. Residues 175–196 and 208–217 have low coordinate RMSD over the structure ensemble and mean S^2^ values 0.766 and 0.837 respectively, indicating only modest internal motion. The second cysteine in each cluster, C179 and C191, has a lower S^2^ value, 0.684 and 0.679 respectively, indicating more internal motion compared to other residues in the well-structured region. Both cysteine clusters have rapid internal motion in the low ps time scale, while the region around cluster one also undergoes chemical exchange.

### Comparison to type 1 CXXC domains

**Fig. 3:**
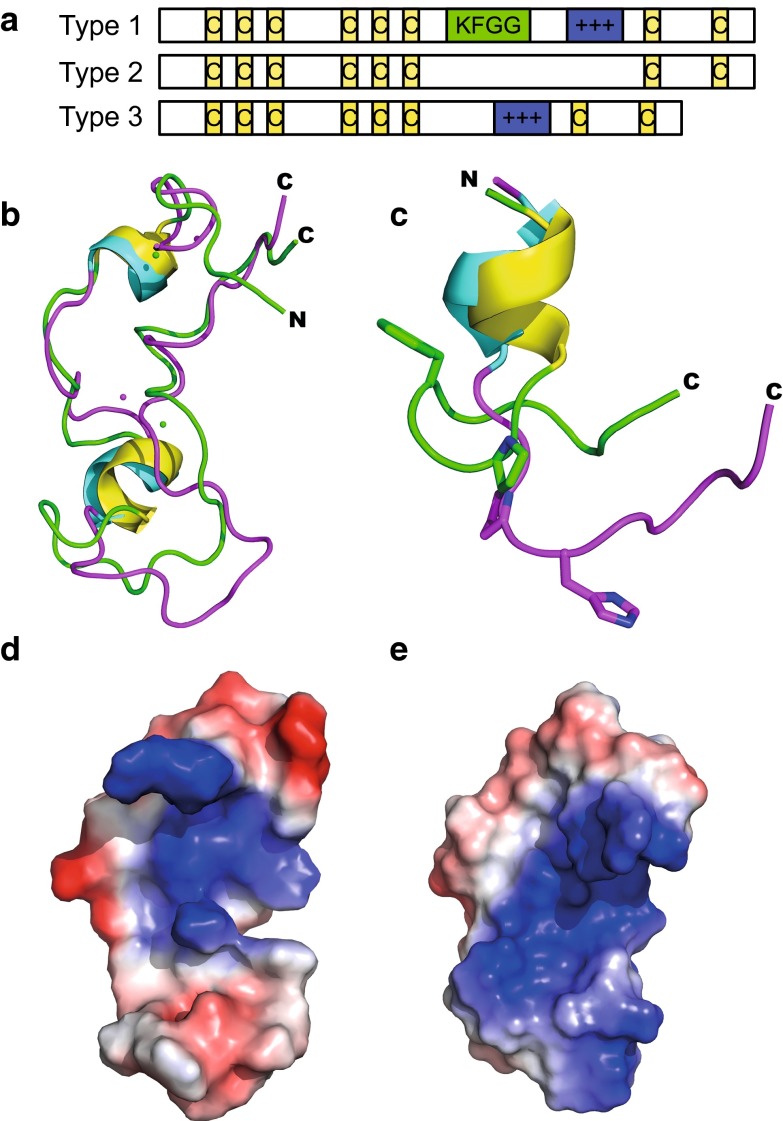
**a** Schematic of CXXC domains showing the features of the three main types. The conserved Cys residues are indicated in yellow while the DNA-binding motifs are indicated in *blue with plus signs*. The KFGG sequence that rigidifies the loop of type 1 domains is *highlighted*. **b** Comparison of the structures of hMBD1 CXXC1 (*magenta* and *cyan*) and the MLL CXXC domain (*green* and *yellow*) and **c** close up of their loops showing how FXXX locks the loop to the second zinc cluster in MLL. **d**, **e** Comparison of the surface electrostatic potentials of hMBD1 CXXC1 and MLL CXXC, respectively, revealing a net negative charge on the loop region of hMBD1 CXXC1, contrasting with the positively charged DNA binding face for MLL CXXC

The solution structure of hMBD1 CXXC1 presented in this paper shows both similarities and differences to the known structures of type 1 CXXC domains (Allen et al. 2006; Cierpicki et al. 2010; Song et al. 2011; Xu et al. 2011) (Fig. 3b). The two zinc coordinating cysteine clusters of the CXXCXXC motifs form a common fold that is likely to be common to all other CXXC domains. However, hMBD1 CXXC1 lacks the KFGG motif that forms a small helix after the second CXXCXXC sequence present in type 1 non-methyl CpG binding CXXC domains. In hMBD1 CXXC1, the corresponding sequence, ^199^PHDV^202^, has a significant effect on the structure of the loop region since P199 does not allow the hydrophobic side chain of H200 to pack up against β protons of C194 to form a helix, resulting in an elongated loop (Fig. 3c). In xDNMT1 CXXC the KFGG sequence is preceded by a lysine rather than the proline in the MLL CXXC domain and the solution structure ensemble of xDNMT1 CXXC (Thomson and Smith, unpublished) reveals a less well defined loop region than hMLL CXXC, implying that the proline preceding the KFGG sequence may be required for loop rigidity. Mutation of hMLL CXXC residues K1178 or F1179 to alanine significantly reduced its ability to bind a 12 bp single CpG DNA in a gel shift assay (Allen et al. 2006) indicating that the precise and ordered structure of the loop may be required for efficient DNA binding.

DNA binding domains are generally evolved to contain positively charged amino acids through which they contact the negatively charged DNA phosphate backbone (Jones et al. 2003) while the shape of the DNA binding face is often complementary to the DNA duplex surface (Tsuchiya et al. 2004). The surface charge plot of hMBD1 CXXC1 (Adaptive Poisson–Boltzmann Solver plugin, PyMol) reveals a positive charge surrounding the first CXXCXXC motif while the second CXXCXXC motif and loop region has an overall negative charge (Fig. 3d). This contrasts with the positively charged face of DNA binding loop of the hMLL CXXC domain (Fig. 3e). This is not surprising as hMBD1 CXXC1 lacks the conserved, positively charged residues found in type 1 domains required for binding to CG nucleotides.

The function of hMBD1 CXXC1 is currently unknown although it has been shown to bind residues 250–337 of Ring1b (a component of the Polycomb group (PcG) multi-protein PRC1 complex). Since hMBD1 CXXC3 does not interact with Ring1b (Sakamoto et al. 2007), the different loop region of hMBD1 CXXC1 may be involved in the protein interaction with Ring1b.
